# Bioenergetic Impairment in Congenital Muscular Dystrophy Type 1A and Leigh Syndrome Muscle Cells

**DOI:** 10.1038/srep45272

**Published:** 2017-04-03

**Authors:** Cibely C. Fontes-Oliveira, Maarten Steinz, Peter Schneiderat, Hindrik Mulder, Madeleine Durbeej

**Affiliations:** 1 Unit of Muscle Biology, Department of Experimental Medical Science, Lund University, Lund, Sweden; 2 Friedrich-Baur-Institute, Department of Neurology, Ludwig-Maximilians-University of Munich, Munich, Germany; 3 Unit of Molecular Metabolism, Department of Clinical Sciences, Lund University Diabetes Centre, Malmö University Hospital, Malmö, Sweden

## Abstract

Skeletal muscle has high energy requirement and alterations in metabolism are associated with pathological conditions causing muscle wasting and impaired regeneration. Congenital muscular dystrophy type 1A (MDC1A) is a severe muscle disorder caused by mutations in the *LAMA2* gene. Leigh syndrome (LS) is a neurometabolic disease caused by mutations in genes related to mitochondrial function. Skeletal muscle is severely affected in both diseases and a common feature is muscle weakness that leads to hypotonia and respiratory problems. Here, we have investigated the bioenergetic profile in myogenic cells from MDC1A and LS patients. We found dysregulated expression of genes related to energy production, apoptosis and proteasome in myoblasts and myotubes. Moreover, impaired mitochondrial function and a compensatory upregulation of glycolysis were observed when monitored in real-time. Also, alterations in cell cycle populations in myoblasts and enhanced caspase-3 activity in myotubes were observed. Thus, we have for the first time demonstrated an impairment of the bioenergetic status in human MDC1A and LS muscle cells, which could contribute to cell cycle disturbance and increased apoptosis. Our findings suggest that skeletal muscle metabolism might be a promising pharmacological target in order to improve muscle function, energy efficiency and tissue maintenance of MDC1A and LS patients.

Skeletal muscle is the largest organ in the human body and is used to respond to a broad range of functional demands in each animal species. It represents approximately 50% of the total body weight and plays a central role in whole-body metabolism^1^. For normal function, skeletal muscle critically depends on mitochondrial ATP production through oxidative phosphorylation (OXPHOS), which is fuelled by tricarboxylic acid cycle through glucose/glycolysis, and fatty acids/β-oxidation^2^. Thus, in order to preserve muscle mass and prevent muscle atrophy it is important to maintain the energy balance^3^. Impairment of muscle function due to mitochondrial abnormalities is linked to several pathological conditions such as cancer cachexia, obesity and ageing^4^,^5^,^6^,^7^, but mitochondrial function remains poorly characterized in muscular dystrophy^6^,^8^.

Congenital muscular dystrophy type 1A (MDC1A) is an autosomal recessive disorder caused by mutations in the human *LAMA2* gene, encoding the α2 subunit of laminin-211^8^. Severe hypotonia, progressive muscle weakness and wasting, joint contractures, gravely impaired motor ability and respiratory failure characterize this disorder, which causes great difficulty in daily life and often leads to premature death^8^,^9^. A complex pathology is seen in MDC1A, which results from the dysregulation of many cellular mechanisms. Laminin α2 chain is expressed in the basement membrane surrounding muscle fibres and is attached to muscle cells via integrin α7β1 and dystroglycan interactions. Consequently, absence or reduction of laminin α2 chain leads to altered extracellular matrix expression and dysregulation of integrin α7β1 and dystroglycan-mediated signalling pathways^8^. Apart from this primary defect, several secondary manifestations such as increased apoptosis, enhanced proteasome and autophagic activity, extensive inflammation and pathological fibrosis have been identified^10^,^11^,^12^,^13^,^14^. Many of these disease driving mechanisms have been targeted with success in mouse models for MDC1A^10^,^11^,^12^,^13^,^14^. Still, the clinical appliance of most of these approaches is years away^8^.

Leigh syndrome (LS), primarily described as a subacute necrotizing encephalomyelopathy in 1951, is a neurometabolic disease caused by mutations in genes related to mitochondrial function^15^. LS has a prevalence of 1 per 40,000 live births and is considered as the most common mitochondrial disease in children. The causes are heterogenic and more than 75 disease genes have been identified^16^. One group of mutations is associated with a lack-of-function of the OXPHOS complex IV, also called cytochrome c oxidase (COX). Mutations in *SURF1* (surfeit locus protein 1) are the most common cause of lack-of-function of COX in LS patients^17^,^18^. SURF1 is a nuclear-encoded small hydrophobic protein, localized to the mitochondrial inner membrane and involved in the initial assembly of the 13 subunits of the COX^19^. Patients with SURF1-associated LS (Surf1-LS) present neurodevelopmental regression, hypotonia, spasticity, movement disorders (including chorea), cerebellar ataxia, and peripheral neuropathy^17^. Just like MDC1A, the prognosis of Surf1-LS is poor with a life expectancy reduced to only a few years^17^. Other common characteristics of the diseases include muscle weakness that leads to hypotonia and respiratory weakness, peripheral neuropathy, and epileptic seizures. Right now, there is no effective treatment available for either LS or MDC1A.

In order to unravel novel molecular mechanisms underlying MDC1A, we recently performed a quantitative proteomic analysis of affected muscles in the *dy*^*3K*^*/dy*^*3K*^ mouse model of the disease^20^. A majority of the differentially expressed proteins were found to be involved in various metabolic processes including glycolysis, fatty acid β-oxidation, tricarboxylic acid cycle, respiratory electron transport and oxidative phosphorylation. However, whether a similar metabolic crisis is detected in MDC1A patient cells remains unknown. More importantly, it has been demonstrated that loss of matrix attachment in epithelial cells leads to metabolic stress characterized by reduced nutrient uptake, decreased ATP production and increased levels of reactive oxygen species^21^. Therefore, we hypothesize that the extracellular matrix also regulates cellular metabolism in skeletal muscle cells and that laminin α2 chain-detached skeletal muscle is metabolically compromised. Mitochondrial function has not been thoroughly studied in Surf1-LS muscle cells either, but considering the nature of underlying mutations, we expected that it would be altered. Conversely, increased apoptosis and enhanced proteasome activity have been identified as disease drivers in MDC1A^11^,^12^,^13^, but have not been studied in LS myogenic cells. Hence, we have here characterized apoptosis, proteasome activity and the bioenergetic profile in human muscle cells from MDC1A and Surf1-LS patients.

## Results

### Altered expression of metabolism-related genes in MDC1A and Surf1-LS muscle cells

We recently performed a proteomic analysis of skeletal muscle from the *dy*^3K^*/dy*^*3K*^ mouse model of MDC1A and confirmed differential expression of selected proteins by other means. We found that a large portion of differentially expressed proteins are involved in various metabolic processes^20^. To analyse if similar metabolic alterations are present in human, we analysed the expression of selected orthologue genes in muscle cells from MDC1A patients. In addition, we analysed muscle cells from Surf1-LS patients, which we hypothesized would have an altered metabolic gene expression profile. Genes related to glycolysis, tricarboxylic acid cycle, and respiratory electron transport/oxidative phosphorylation were analysed and included: *PFK*, encoding 6-phosphofruktokinase; *PYGM*, encoding glycogen phosphorylase, muscle form; *PGAM2*, encoding phosphoglycerate mutase 2; *PDHA1*, encoding pyruvate dehydrogenase alpha1; *IDH3*, encoding isocitrate dehydrogenase 3; *SDHA*, encoding succinate dehydrogenase complex flavoprotein subunit A; *ATP5I*, encoding ATP synthase, H^+^ transporting, mitochondrial F0 complex subunit E; *NDUFS2*, encoding NADH dehydroygenase (ubiquinone) Fe-S protein 2; *NDUFA8*, encoding NADH:ubiquinone oxidoreductase subunit A8; *PGC1α*, encoding peroxisome proliferator-activated receptor gamma coactivator 1-alpha and finally *ANT1*, encoding adenine nucleotide translocator 1 (for more details, see Table S1).

**Figure 1:**
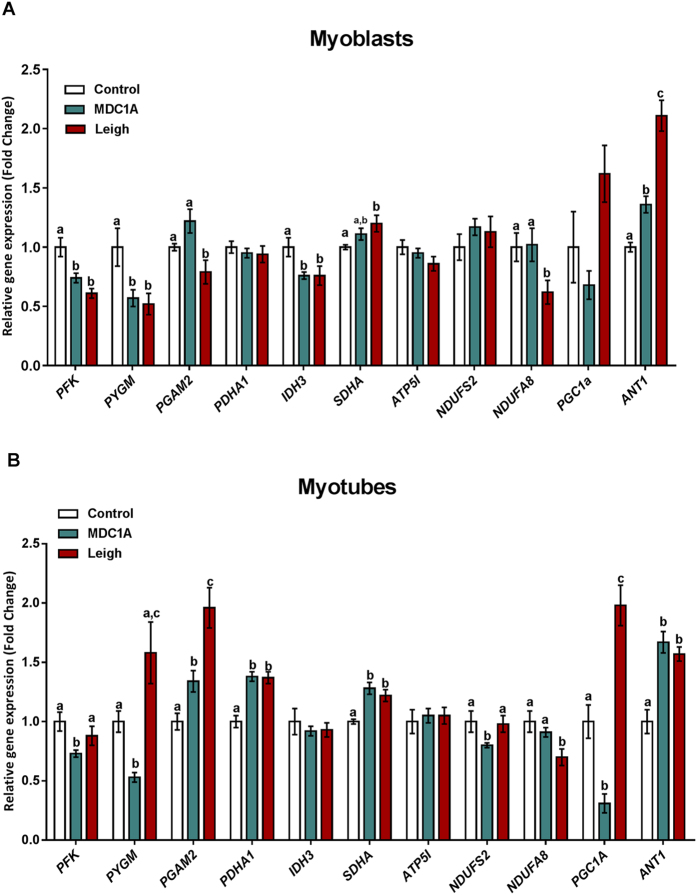
Pattern of gene expression in MDC1A and Surf1-LS myogenic cells. Results represent differences in gene expression in human muscle cells (myoblasts (**A**) and myotubes (**B**) as indicated) among control subjects, MDC1A and Surf1-LS patient groups. For further details, see Methods section and Table S1. *PFK*: 6-phosphofruktokinase; *PYGM:* glycogen phosphorylase, muscle form; *PGAM2:* phosphoglycerate mutase 2; *PDHA1:* pyruvate dehydrogenase alpha1; *IDH3:* isocitrate dehydrogenase 3; *SDHA:* succinate dehydrogenase complex flavoprotein subunit A; *ATP5I:* ATP synthase, H^+^ transporting, mitochondrial F0 complex subunit E; *NDUFS2:* NADH dehydroygenase (ubiquinone) Fe-S protein 2; *NDUFA8:* NADH:ubiquinone oxidoreductase subunit A8; *PGC-1α:* PPAR co-activator 1a; *ANT1:* adenine nucleotide translocator 1. Columns represent mean values and bars SE. Results are expressed as a fold change of controls. Statistical significance was assessed by one-way ANOVA followed by Duncan’s post hoc test. *p* < 0.05 values were considered as statistically significantly different from each other. Letters a, b and c were used to express the differences among groups and columns with the same letter are not significantly different.

Expression of *PFK, PYGM, IDH3*, and *ANT1* was significantly dysregulated in myoblasts from MDC1A and Surf1-LS patients compared to myoblasts from control subjects (Fig. 1A). Additionally, expression of *PGAM2, SDHA* and *NDUFA8* was dysregulated in Surf1-LS myoblasts but remained unaltered in MDC1A myoblasts (Fig. 1A). Subsequently, *PGAM2, PDHA1, SDHA* and *ANT1* were upregulated in both MDC1A and Surf1-LS myotubes (Fig. 1B).

Some genes, such as *NDUFA8, PGC1α* and *ANT1* were similarly regulated in both proliferative and differentiated stages, even though the differences in *PGC1α* expression were not statistically significant in myoblasts (Fig. 1A). In contrast, *PDHA1* gene expression was not altered in MDC1A and Surf1-LS myoblasts but increased in corresponding myotubes (Fig. 1A and B).

There were also some genes whose expression was not coordinately regulated in MDC1A and Surf1-LS cells. One striking example is *PGC1α*, which was downregulated in MDC1A myoblasts and myotubes but upregulated in corresponding Surf1-LS cells. Another example is *NDUFA8*, which was only downregulated in Surf1-LS cells while its expression was not altered in MDC1A myogenic cells (Fig. 1A and B).

In summary, we show a dysregulated gene expression pattern of metabolism-related genes in MDC1A and Surf1-LS myogenic cells. Considering these results and previous proteomic data obtained from MDC1A mouse muscle, also demonstrating down- and upregulation of metabolism-related proteins, we hypothesise that metabolic function should be altered in MDC1A and Surf1-LS myogenic cells.

### Reduced mitochondrial respiration and ATP production in MDC1A and Surf1-LS muscle cells

**Figure 2:**
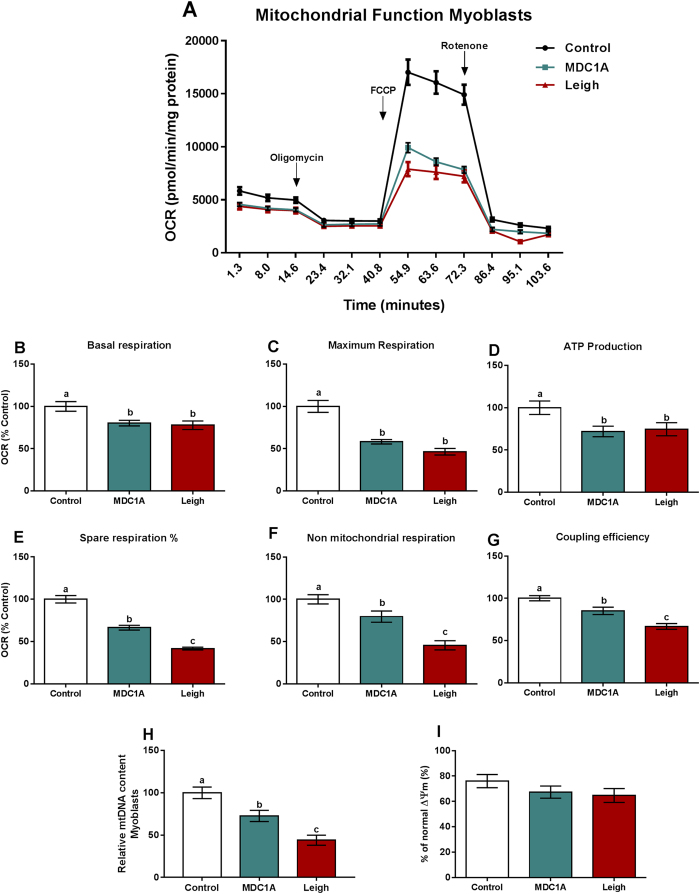
Impaired mitochondrial function in myoblasts from MDC1A and Surf1-LS patients. (**A**) OCR of control, MDC1A and Surf1-LS myoblasts in response to 5 μM oligomycin, 1 μM FCCP and 1 μM rotenone. For further details see methods section. Decreased oxygen consumption was observed in MDC1A and Surf1-LS myoblasts compared to control cells. (**B**) Basal respiration; (**C**) Maximum Respiration; (**D**) ATP Production; (**E**) Spare respiration %; (**F**) Non-mitochondrial respiration; (**G**) Coupling efficiency. (**H**) Relative mtDNA content was decreased in MDC1A and Surf1-LS myoblasts compared to control cells. Results are expressed as a fold change of controls. (**I**) FACS measurement of ΔΨm using JC-1. Results are expressed as % cell population with normal ΔΨm. Columns represent mean values and bars SE. n = 3–10 biological replicates per patient and subject. Statistical significance was assessed by one-way ANOVA followed by Duncan’s post hoc test. *p* < 0.05 values were considered as statistically significantly different from each other. Letters a, b and c were used to express the differences among groups and columns with the same letter are not significantly different from another.

**Figure 3:**
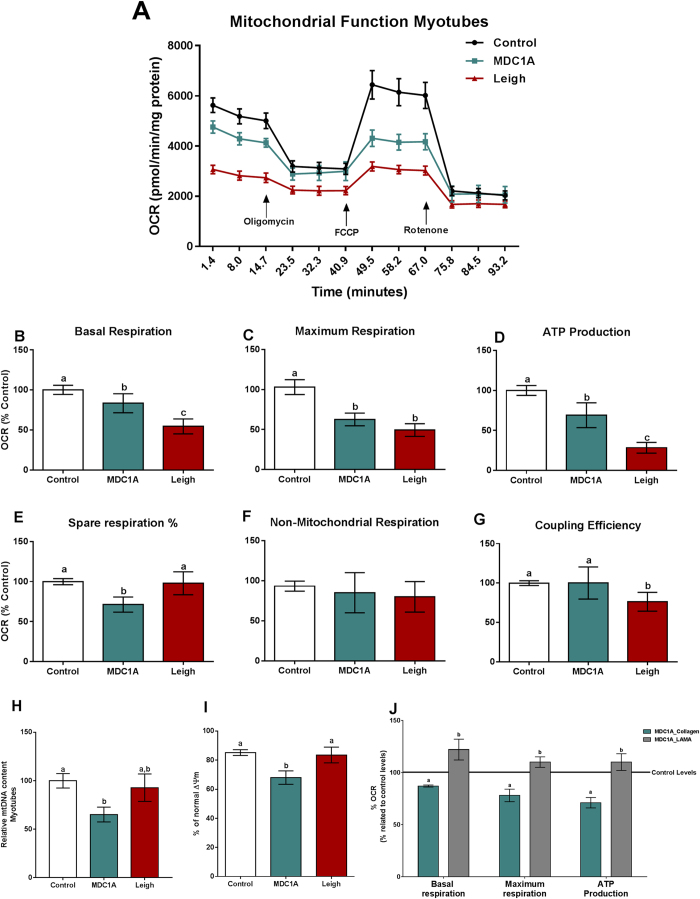
Impaired mitochondrial function in myotubes from MDC1A and Surf1-LS patients. (**A**) OCR of control, MDC1A and Surf1-LS myotubes in response to 5 μM oligomycin, 1 μM FCCP and 1  μM rotenone. For further details, see methods section. Decreased oxygen consumption was observed in MDC1A and Surf1-LS myotubes compared to control cells. (**B**) Basal respiration; (**C**) Maximum Respiration; (**D**) ATP Production; (**E**) Spare respiration %; (**F**) Non-mitochondrial respiration; (**G**) Coupling efficiency. (**H**) Relative mtDNA content was decreased in MDC1A and unaltered Surf1-LS myotubes compared to control cells. (**I**) FACS measurement of ΔΨm using JC-1. Results are expressed as % cell population with normal ΔΨm. (**J**) Mitochondrial function is restored by presence of laminin-211 in MDC1A myotubes. Columns represent mean values and bars SE. n = 3–10 biological replicates per patient and subject. Statistical significance was assessed by one-way ANOVA followed by Duncan’s post hoc test. *p* < 0.05 values were considered as statistically significantly different from each other. Letters a, b and c were used to express the differences among groups and columns with the same letter are not significantly different from another.

In order to assess whether alterations in gene expression were accompanied by functional changes, we next experimentally analysed mitochondrial respiration in MDC1A and Surf1-LS myoblasts and myotubes from human patients. For this purpose, oxygen consumption rate (OCR, defined as the rate of change by which a cell consumes oxygen) was measured in real-time with injections of oligomycin, FCCP and rotenone in order to inhibit ATP synthase, uncouple respiration and inhibit complex I of OXPHOS system, respectively^22^,^23^. Notably, we found significantly reduced OCR, which reflects decreased basal respiration, maximum respiration and ATP production in MDC1A and Surf1-LS myoblasts and myotubes compared with controls (Figs 2 A–D and 3 A–D). Spare respiration was also reduced in MDC1A and Surf1-LS myoblasts and in MDC1A myotubes (Figs 2E and 3E). Non-mitochondrial respiration was decreased in MDC1A and Surf1-LS myoblasts but not in corresponding myotubes (Figs 2F and 3F) and coupling efficiency was also diminished in MDC1A myoblasts and in Surf1-LS myotubes (Figs 2G and 3G). Altogether, these data indicate that oxidative phosphorylation is severely impaired in MDC1A and Surf1-LS myogenic cells.

This impairment can be due to deficiencies in mitochondria content, reduced mitochondria function, or both. It is well known that mitochondrial biogenesis is controlled by nuclear genes, such as *PGC1α*^24^. In fact, *PGC1α* expression was reduced in MDC1A myotubes and increased in Surf1-LS myotubes, indicating that mitochondrial content could be different in the two cell types (Fig. 1A and B). Thus, we next measured the mtDNA content as it is an indicator of mitochondrial content^25^ and evaluated the integrity of the mitochondrial membrane. The relative mtDNA content was decreased in MDC1A and Surf1-LS myoblasts (Fig. 2H) and was accompanied by a slight reduction of the cell population with polarized mitochondria in MDC1A myoblasts, but this was not statistically significant (Fig. 2I). In MDC1A myotubes, we found an approximate 40% reduction of the relative mtDNA content but noted normal mtDNA content in Surf1-LS myotubes (Fig. 3H). Similarly, *mt-COX1* expression analysis confirmed these results (Figure S1). Additionally, the percentage of cell population with normal mitochondrial membrane potential (ΔΨm) was reduced in MDC1A myotubes but not altered in Surf1-LS myotubes (Fig. 3I). In summary, these results indicate that absence of laminin α2 chain leads to downregulated *PGC1α* expression, which impairs mitochondrial biogenesis, causing a reduction of mitochondrial content that finally leads to a bioenergetic inefficiency in myoblasts and myotubes from MDC1A patients.

To confirm that the metabolic alterations were due to deficiency of laminin-211 in MDC1A cells, we cultured the MDC1A myotubes in plates coated with recombinant laminin-211 (Fig. 3J). Indeed, basal respiration, maximum respiration and ATP production as well as basal mitochondrial respiration and maximal mitochondrial respiration capacity were normalized to control levels in the presence of laminin-211 (Fig. 3J).

In Surf1-LS cells, on the other hand, the impaired OXPHOS system is related to mitochondria dysfunction, rather than deficiency in mitochondrial content and this mitochondria dysfunction cannot be overcome by the endogenously increased *PGC1α* expression.

### Increased glycolysis in MDC1A and LS muscle cells

**Figure 4:**
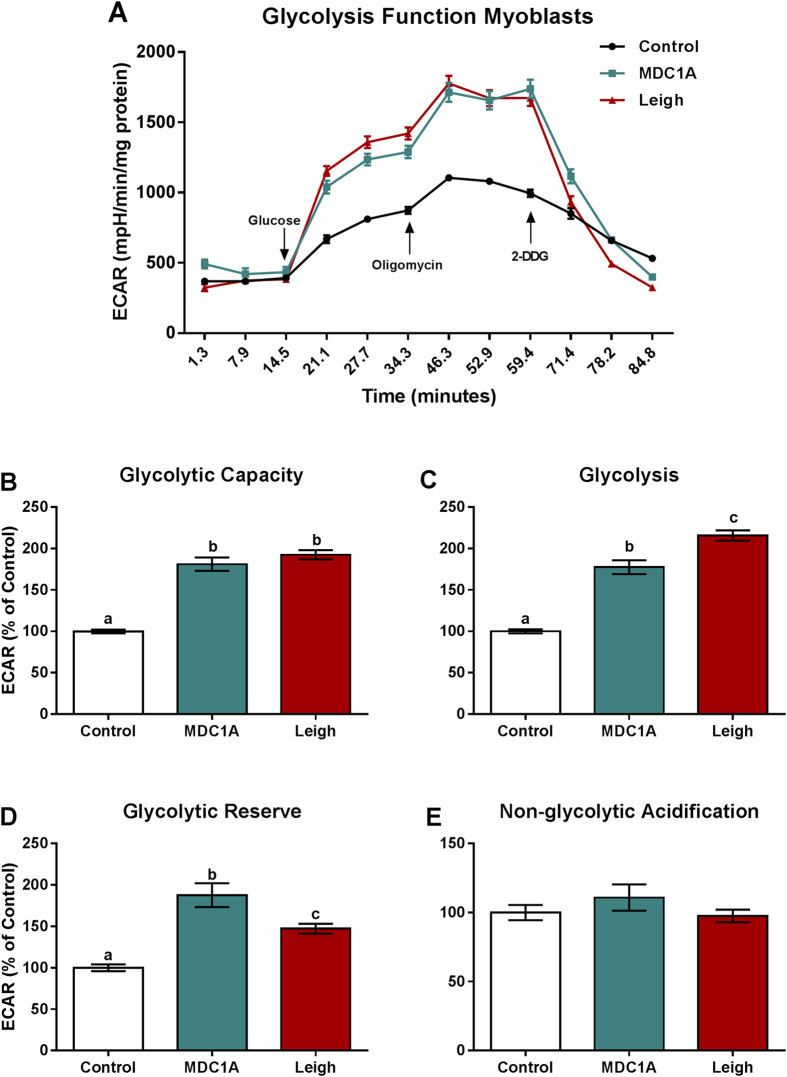
Increased glycolytic function in myoblasts from MDC1A and Surf1-LS patients. (**A**) ECAR of control, MDC1A and Surf1-LS myoblasts in response to 10 μM glucose, 2.5 μM oligomycin and 50 mM 2DDG. Increased extracellular acidification was observed in MDC1A and Surf1-LS myoblasts compared to control cells. (**B**) Glycolytic Capacity; (**C**) Glycolysis; (**D**) Glycolytic reserve; (**E**) Non-glycolytic acidification. For further details, see methods section. Columns represent mean values and bars SE. n = 10 biological replicates per patient and subject. Statistical significance was assessed by one-way ANOVA followed by Duncan’s post hoc test. *p* < 0.05 values were considered as statistically significantly different from each other. Letters a, b and c were used to express the differences among groups and columns with the same letter are not significantly different from another.

**Figure 5:**
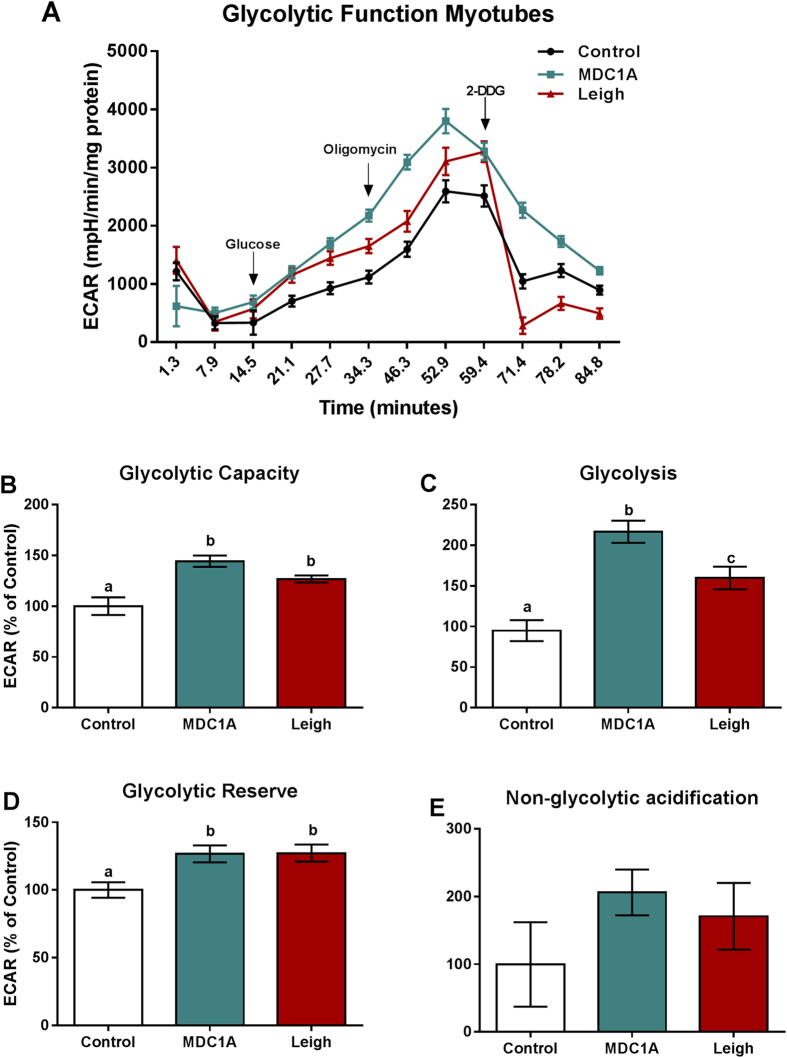
Increased glycolytic function in myotubes from MDC1A and Surf1-LS patients. (**A**) ECAR of control, MDC1A and Surf1-LS myotubes in response to 10 μM glucose, 2.5 μM oligomycin and 50 mM 2DDG. Increased extracellular acidification was observed in MDC1A and Surf1-LS myotubes compared to control cells. (**B**) Glycolytic Capacity; (**C**) Glycolysis; (**D**) Glycolytic reserve; (**E**) Non-glycolytic acidification. For further details, see methods section. Columns represent mean values and bars SE. n = 10 biological replicates per patient and subject. Statistical significance was assessed by one-way ANOVA followed by Duncan’s post hoc test. *p* < 0.05 values were considered as statistically significantly different from each other. Letters a, b and c were used to express the differences among groups and columns with the same letter are not significantly different from another.

In order to quantify changes in the glycolytic metabolism, we next measured extracellular acidification rate (ECAR, defined as the rate of change in proton excretion from the cell) in real-time with injections of glucose, oligomycin and 2-DG (for more details, see methods section. Surprisingly, we found that ECAR was significantly higher in MDC1A myoblasts and myotubes compared to control myogenic cells (Figs 4A and 5A). Likewise, ECAR was significantly higher in Surf1-LS myoblasts and myotubes compared to control cells (Figs 4A and 5A). Glycolysis, glycolytic capacity and glycolytic reserve were all significantly increased in MDC1A and Surf1-LS myogenic cells, whereas non-glycolytic acidification remained unchanged (Figs 4B–E and 5B–E). All in all, these data suggest that the impaired OXPHOS in MDC1A and Surf1-LS myogenic cells leads to an increased dependence on glycolysis for ATP production.

### Reduced fatty acid oxidation in MDC1A and Surf1-LS muscle cells

**Figure 6:**
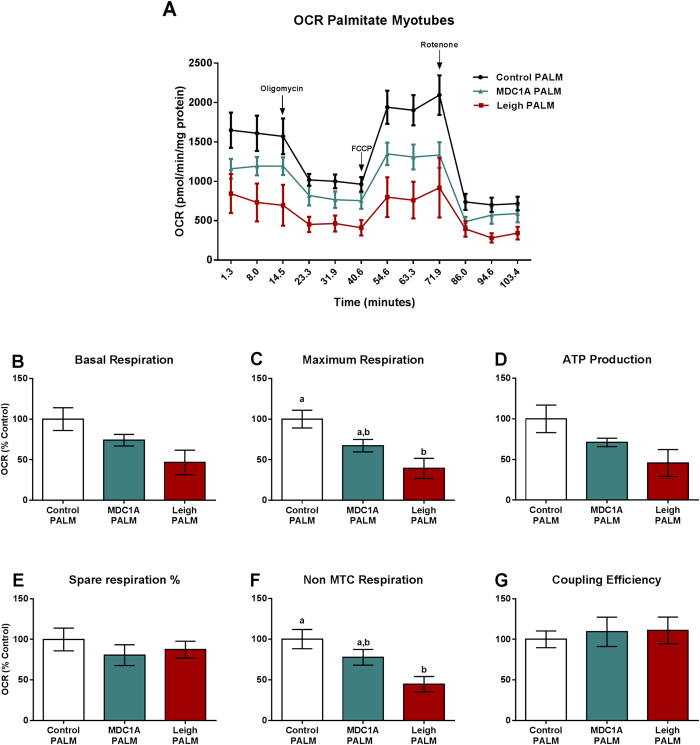
Decreased FAO uptake in myotubes from MDC1A and Surf1-LS patients. (**A**) OCR of control, MDC1A and Surf1-LS myoblasts in response to 5 μM oligomycin, 5 μM, 1 μM FCCP and 1 μM rotenone. Decreased exogenous FA utilization was observed in MDC1A and Surf1-LS cells compared to control cells. For further details, see methods section. (**B**) Basal respiration; (**C**) Maximum Respiration; (**D**) ATP Production; (**E**) Spare respiration %; (**F**) Non-mitochondrial respiration; (**G**) Coupling efficiency. Columns represent mean values and bars SE. n = 3 biological replicates per patient and subject. Statistical significance was assessed by one-way ANOVA followed by Duncan’s post hoc test. *p* < 0.05 values were considered as statistically significantly different from each other. Letters a, b and c were used to express the differences among groups and columns with the same letter are not significantly different from another.

In order to evaluate utilization of external fatty acids (FA) in MDC1A and Surf1-LS muscle cells, we assessed OCR (after starving cells for 12 h) just after adding palmitate to the media. Figure 6 shows decreased maximum mitochondrial respiration in MDC1A and Surf1-LS myotubes, compared with myotubes from control subjects indicating an impairment of exogenous FA uptake. Basal respiration and non-mitochondrial respiration were significantly decreased in Surf1-LS myotubes as well, and the same tendency was observed in MDC1A myotubes (Fig. 6). No difference was detected in MDC1A and Surf1-LS myoblasts (data not shown).

### Disturbances in cell cycle population and apoptosis

**Figure 7:**
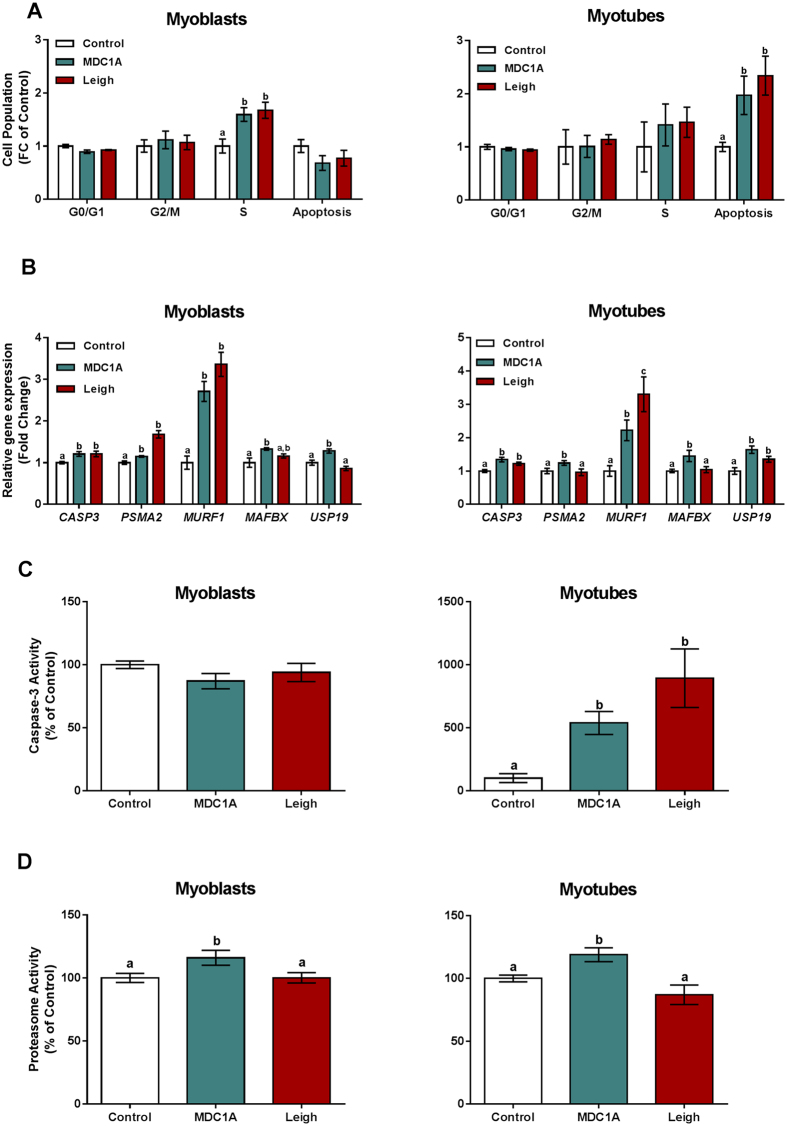
Cell cycle, apoptotic and proteolytic profiles in MDC1A and Surf1-LS muscle cells. (**A**) Cell distribution in the different phases of the cell cycle. (**B**) Differences in gene expression of *CASP3, PSMA2, MURF1, MAFBX* and *USP19* in MDC1A and Surf1-LS myoblasts and myotubes compared to control. (**C**) Caspase-3 enzymatic activity was increased in myotubes from MDC1A and Surf1-LS patients compared to control cells. (**D**) 20S proteasome enzymatic activity was increased in both myoblasts and myotubes from MDC1A patients but was unchanged in Surf1-LS muscle cells compared to control. Results are expressed as a fold change of controls. Columns represent mean values and bars SE. Statistical significance was assessed by one-way ANOVA followed by Duncan’s post hoc test. *p* < 0.05 values were considered as statistically significantly different from each other. Letters a, b and c were used to express the differences among groups and columns with the same letter are not significantly different from another.

Considering the altered metabolic profile in MDC1A and Surf1-LS muscle cells, we next analysed if the bioenergetic impairment could influence cell cycle populations, affecting cell proliferation, differentiation and viability. We analysed the cell cycle profile and quantified the cell populations in MDC1A and Surf1-LS myoblasts and myotubes. Among all cell cycle phases (G0/G1, S and G2/M), we observed increased S population in MDC1A and Surf1-LS myoblasts. A similar tendency was seen in myotubes, but the difference was not statistically significant (Fig. 7A). This profile was accompanied by an increased apoptotic cell population in MDC1A and Surf1-LS myotubes compared with control cells. These data indicate that disturbances in the cell cycle could be linked to impairment of cell differentiation, leading to apoptosis (Fig. 7A).

### Increased apoptosis and proteasome activity in MDC1A and Leigh muscle cells

Caspase-3 activation has been shown to be involved in impairment of satellite cell renewal and participate in apoptotic events during muscle repair in mitochondrial diseases^26^,^27^. Moreover, enhanced apoptosis and proteasome activity have been implicated in the pathology of MDC1A^13^,^28^. In order to confirm whether these events occurred in the muscle cells from MDC1A and Surf1-LS patients we assessed gene expression and enzymatic activity of caspase-3 and 20S proteasome. We observed an increased *Caspase-3* mRNA expression in MDC1A and LS myoblasts and myotubes (Fig. 7B). Caspase-3 activity was concomitantly enhanced in MDC1A and Surf1-LS myotubes but not in corresponding myoblasts, indicating that the apoptotic events are present during the differentiation process in these diseases (Fig. 7C). The mRNA expression of proteasome-related genes such as *20S core particle subunit α2 (PSMA2*), *MuRF1, MAFbx* and *USP19* was significantly augmented in MDC1A myoblasts and myotubes (Fig. 7B). Similarly, proteasome activity was escalated in MDC1A myoblasts and myotubes (Fig. 7D). The expression of some proteasome-related mRNAs was increased in Surf1-LS myoblasts and myotubes (Fig. 7B) but the activity of 20S proteasome was not significantly altered in myoblasts or myotubes from Surf1-LS patients (Fig. 7D). Conclusively, these data show that the MDC1A cells recapitulate pathological changes that have been reported previously, but that the proteasome activity is not enhanced in Surf1-LS muscle cells.

## Discussion

A bioenergetic inefficiency can break the balance between anabolic and catabolic processes in skeletal muscle and lead to loss of muscle mass and muscle strength. Indeed, previous studies have described impaired metabolism in neuromuscular diseases^29^ but muscular dystrophies remain poorly characterized in this respect.

Our experimental data showed a dysregulated expression pattern of several genes related to metabolism in MDC1A and Surf1-LS muscle cells. We observed an increased expression of *PGAM2* in myotubes of MDC1A and Surf1-LS patients, and *PGAM2* encodes an enzyme involved in the energy generating phase of glycolysis. Okuda *et al*. observed that *PGAM2* overexpression was related to decreased expression of glycolytic enzymes, alteration of metabolites involved in glycolysis and the tricarboxylic acid cycle, decreased mitochondrial respiration and increased mitochondrial ROS production in cardiomyocytes^30^. In fact, we observed a decreased *PFK* expression in MDC1A cells, and *PFK* encodes an important rate-limiting enzyme in glycolysis, upstream of PGAM2 activity. IDH3, a NAD^+^-dependent enzyme, is responsible for isocitrate conversion into α-ketoglutarate in the tricarboxylic acid cycle. IDH3 activity is stimulated by Ca^2+^, as well as by increased ADP/ATP and NAD^+^/NADH concentration ratios, supporting its function in the oxidative direction of the tricarboxylic acid cycle^31^. In myoblasts, a 34% decreased expression of *IDH3* was observed but no changes were observed in myotubes of MDC1A and Surf1-LS patients. Since IDH3 plays an important function in the oxidative direction of the Krebs cycle, the decreased *IDH3* expression could indicate a change of metabolism in MDC1A and Surf1-LS myoblasts. Based on our previous proteomic studies^20^, we expected to find genes related to the OXPHOS system differentially expressed. In fact, a 20% reduced expression of *NDUFS2* in MDC1A myotubes and a 30% reduction of *NDUFA8* expression in Surf1-LS myotubes was observed. *NDUFS2* and *NDUFA8* encode the NADH dehydroygenase (ubiquinone) Fe-S protein 2 and NADH: ubiquinone oxidoreductase subunit A8, respectively. Both are parts of the nicotinamide adenine dinucleotide (NADH): ubiquinone oxidoreductase enzyme (complex I) of OXPHOS system. It is known that some cases of LS are related to deficiency of nuclear-encoded subunits of complex I^32^. Altogether, our data suggest that Surf1-deficiency could somehow influence not just only COX activity but also other OXPHOS complexes in human myogenic cells.

An increased *ANT1* expression was observed in muscle cells from MDC1A and Surf1-LS patients. ANT1 is the predominant isoform present in skeletal muscle and although not normally included as a part of the OXPHOS system, it is a key protein regulating the mitochondrial ATP/ADP flux. It is known that there is a striking link between ANT1 and mitochondrial uncoupling in skeletal muscle, which refers to the dissociation of mitochondrial respiratory chain activity from ATP synthesis in OXPHOS, due to a proton leakage across the inner mitochondrial membrane^33^. It has been demonstrated that ANT1 is overexpressed in muscle of facioscapulohumeral muscular dystrophy (FSHD) patients^34^. FSHD is characterized by the adult onset of progressive weakness in muscles of the face, shoulders, feet and hips. FSHD patients show a significantly elevated *ANT1* gene expression and increased amounts of ANT1 protein in skeletal muscle, resulting in mitochondrial dysfunction associated with increased oxidative stress^35^. Moreover, ANT1 expression is also enhanced in muscles from patients with Duchenne muscular dystrophy^35^ and has been shown to be correlated to mitochondrial alterations in atrophy caused by immobilization^36^ and cancer cachexia *in vivo*^4^. Altogether, our results may suggest that ANT1 overexpression could contribute to the bioenergetic impairment in both MDC1A and Surf1-LS muscle.

Another gene that we analysed was *PGC1α*, encoding a key nuclear receptor co-activator for mitochondrial biogenesis and formation/maintenance of slow twitch fibres in skeletal myocytes^37^. Here, we observed divergent results between MDC1A and Surf1-LS myogenic cells. A decreased expression of *PGC1α* was observed in myoblasts and myotubes from MDC1A patients, whereas we noted an increased expression in Surf1-LS myoblasts and myotubes. These results could be due to the different origin of the two diseases as MDC1A is an extracellular matrix-related disease whereas Surf1-LS is a mitochondrial disorder *per se*. Indeed, altered *PGC1α* expression can have distinct consequences in different pathological conditions. For example, overexpression of *PGC1α* in mice markedly decreased the ATP content in skeletal muscle and resulted in myopathy at 25 weeks of age, leading to muscle atrophy and lipid accumulation^38^. Moreover, an increased expression of *PGC1α* was observed in cancer cachectic animals and related to alterations in mitochondria morphology and function^4^,^39^. On the other hand, a decreased mitochondrial content, impaired mitochondria function and enhanced apoptotic susceptibility was observed in mice lacking *PGC1α*^40^*. PGC1α* is also related to protective effects on muscle mass under hindlimb unloading, preventing catabolism system activation in mice^41^. Some studies demonstrated increased *PGC1α* expression related to improvement of mitochondrial function in skeletal muscle of obese rats treated with polyunsaturated fatty acid^42^ and in dystrophin-deficient *mdx* muscle treated with the anti-diabetic drug metformin^43^. Our data corroborate the findings of Pullian *et al*., who observed a link between decreased COX activity and increased expression *PGC1α* in *Surf1*^*−/−*^ mice, which can be a stress response in order to confer protective effects on cellular homoeostasis^19^. In fact, Viscomi *et al*.^44^ observed increased mitochondrial biogenesis and activities of OXPHOS components when *PGC1α* was overexpressed in *Surf1*^*−/−*^ mice. In summary, our results confirm that *PGC1α* can be differentially expressed depending on the muscle disease.

Based on the gene expression profile, we hypothesized that the cellular bioenergetic system would be impaired in MDC1A and Surf1-LS myoblasts and myotubes. Indeed, when we assessed the function of the OXPHOS system and glycolysis in MDC1A and Surf1-LS cells, modulating those processes in real-time, we observed an energetic impairment of MDC1A and Surf1-LS muscle cells. Accordingly, the reduced mitochondrial function was accompanied by enhanced glycolytic activity. Moreover, a decrease of exogenous FA utilization in Surf1-LS myotubes was observed. All in all, these striking changes in metabolism were noted in both MDC1A and Surf1-LS and our results indicate that mitochondrial dysfunction would impair not only the skeletal muscle function but also interfere with the muscle regeneration and repair, since myoblasts are also bioenergetically impaired.

The bioenergetic inefficiency can be due to reduced mitochondria content, diminished mitochondria function, or both^25^. In fact, we observed a reduction of the relative mtDNA content in MDC1A myotubes, corroborating the *PGC1α* gene expression profile. On the other hand, relatively normal levels of mtDNA were observed in Surf1-LS myotubes, indicating that the impairment of the OXPHOS system is related to mitochondria dysfunction, rather than deficiency of mitochondrial content in Surf1-LS.

The consequences of mtDNA stability and content have been studied in some conditions like cancer^45^,^46^ and cardiomyopathies^47^. Furthermore, the therapeutic potential of increasing mtDNA content has been explored. For example, supplementation with butyrate in maternal diet was able to improve mtDNA content and increase mitochondrial activity in skeletal muscle from rat offspring^48^. Also, overexpression of PGC1α ameliorates muscular dystrophy in dystrophin-deficient mice^49^. Hence, it would be interesting to analyze if genetic or pharmacological approaches aimed at increasing mitochondrial biogenesis would have beneficial effects in MDC1A muscle.

The shift towards a high rate of glycolysis in MDC1A and Surf1-LS cells probably occurs in order to compensate for the diminished energy production by impaired mitochondria. It is known that highly proliferative cells, such as cancer cells^50^ and in immune cells^51^ as well, generate ATP in an inefficient fashion, preferentially by utilizing glycolysis rather than oxidative phosphorylation. This phenomenon is described as the Warburg effect or aerobic glycolysis and it has been proposed to facilitate the uptake and incorporation of nutrients needed to produce a new cell^50^. In fact, we also observed a disturbance in the cell cycle population with an increased proliferative state of MDC1A and Surf1-LS myoblasts, which confirms recent findings which associated proliferation of myoblasts *in vitro* with downregulation of OXPHOS and energy storage in ageing^52^. In addition, following differentiation we observed increased apoptosis in myotubes, confirming that bioenergetic impairment may lead to a disturbance in the cell cycle and late apoptosis in MDC1A and Surf1-LS.

Notably, apoptosis and the ubiquitin proteasome system play critical roles in the development of skeletal muscle atrophy^27^,^53^. Increased *caspase-3* gene expression and enzymatic activity in myotubes indicated that apoptotic processes are part of muscle pathology in MDC1A, as previously described^28^ but shown for the first time for Surf1-LS. Also, increased gene expression of components of the ubiquitin-proteasome system and increased enzymatic activity of the 20S proteasome were observed in human MDC1A myoblasts and myotubes, confirming what was previously observed in muscle cells derived from one MDC1A foetus^13^. In Surf1-LS cells we observed increased gene expression of some components of the proteasome system, but this pattern did not reflect the enzymatic activity of the 20S-proteasome. These data indicate that proteolytic events are not part of the Surf1-LS pathology.

Although Surf1-LS and MDC1A myogenic cells from patients present energetic inefficiency, our data suggest that the molecular responses in these pathological conditions are different. The extracellular matrix (ECM) is known to provide physical support to tissues and organs and to maintain cell integrity and allow transduction of molecular signals, which have critical roles in tissues undergoing extensive mechanical stress, like skeletal muscle^54^. However, disturbances in ECM components, such as laminin-211, on fundamental aspects of mitochondrial biology have barely been studied before. The insight into abnormal mitochondrial function in MDC1A has come from studies of calcium handling and mitochondrial permeability transition pore opening. Treatment with an inhibitor of cyclophilin-D, which is a regulatory protein of the permeability transition pore, reduced muscular dystrophy pathology in laminin α2 chain-deficient mice^55^. In addition, it is known that deficiency of collagen VI affects intracellular signalling pathways impairing the mitochondria function, leading to apoptosis in *Col6a1*^−/−^ mice^56^ and other extracellular matrix proteins such as fibronectin and MMP-2 have been demonstrated to influence mitochondrial function^57^. Here, we present the first evidence that laminin α2 chain regulates *PGC1α* expression and mitochondrial content and that deficiency of mitochondrial content leads to bioenergetic impairment in MDC1A myogenic cells. Thus, it is becoming increasingly clear that a relationship exists between the extracellular matrix and mitochondria. However, it remains to be elucidated how these extracellular signals are transduced within muscle fibres.

Considering that LS is a neuropathology linked to mutations in genes encoding OXPHOS components^16^, it may not be surprising that metabolic function is affected in muscle. However, muscle cells from Surf1-LS patients have, to our knowledge, never been metabolically characterized. Our data suggest that the bioenergetic impairment triggers a response, such as increased *PGC1α* expression, in order to supply the energetic demand in Surf1-LS myoblasts. The increased *PGC1α* expression is maintained in myotubes but despite normalization of mitochondrial content, mitochondrial respiration remains compromised in Surf1-LS myotubes. Our results could in fact explain the symptoms of muscle weakness and hypotonia in Surf1-LS patients and are in agreement with a recent study, which demonstrated impaired metabolism in fibroblasts from LS patients with mtDNA mutations^58^. Pharmacological strategies such as treatment with antioxidants would be an interesting approach to improve mitochondrial function and to ameliorate the clinical features in Surf1-LS^59^,^60^.

**Figure 8:**
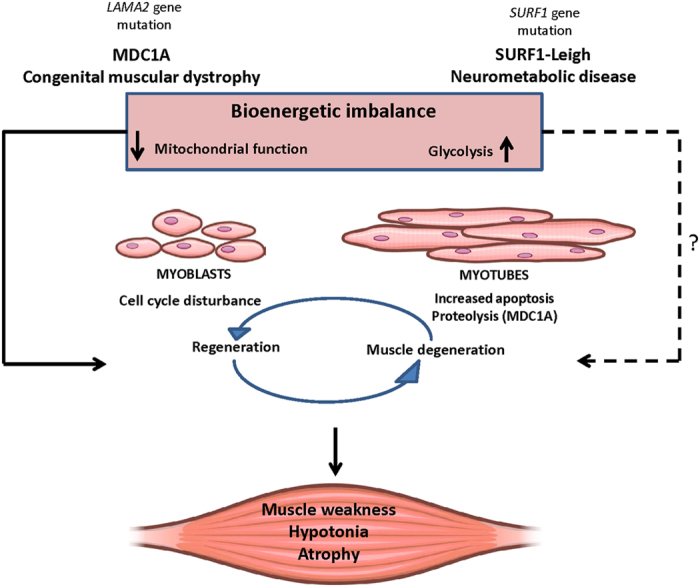
Hypothetical contribution of bioenergetic impairment in MDC1A and Surf1-LS pathologies. Reduced mitochondrial function and enhanced glycolysis could contribute to cell cycle disturbances in myoblasts and increased apoptosis and proteolysis in myotubes. As a consequence, muscle maintenance would be compromised, leading to fibrosis and muscle atrophy.

In summary, our results suggest that in muscular pathological conditions, such as MDC1A and LS, an increase of glycolytic metabolism could be an attempt to compensate for the impairment of mitochondrial function in order to supply the energy demands of the cell. Since this scenario is not ideal for a tissue with high demand of energy production, such as skeletal muscle, this bioenergetic imbalance could cause cell cycle disturbance in myoblasts and increased apoptosis and proteolysis in myotubes. As a consequence, muscle degeneration and perhaps also the regenerative capacity^61^ could be affected, leading to muscle weakness and atrophy in MDC1A, and maybe in Surf1-LS as well (Fig. 8). The present study was performed with primary cells from patients, which is a limitation of our study due to senescence and the changing of molecular characteristics along cell passages^62^. With the advent of immortalized cells from muscular diseases, such as MDC1A^28^, we hope that the molecular processes involved in this phenomenon can be clarified in the future. The bioenergetic impairment as a common denominator between MDC1A and Surf1-LS is an important finding in this study. Thus, independently of the underlying cause, metabolism may be a key system affected in diseases involving skeletal muscle. Moreover, our findings reinforce expert opinions that point out mitochondrial function and content as a key factor to maintain skeletal muscle homeostasis and function. Thus, a normal mitochondrial function reflects quality of life in health and constitute a promising target using pharmacological approaches in pathologies related to muscular dysfunction and atrophy^5^,^7^,^53^,^63^.

## Methods

### Human cells

Muscle primary cells from 3 MDC1A and 3 Surf1-LS patients and 3 normal subjects were provided by Muscle Tissue Culture Collection (MTCC) from University of Munich (http://www.klinikum.uni-muenchen.de/Friedrich-Baur-Institut/de/forschung/muskelbank/, last accessed March 16, 2017). Cells were collected and processed by MTCC in compliance with all applicable laws, rules, regulations and other requirements of any applicable governmental authority, including without limitation those applicable to patient informed consent^64^.

### Cell culture

Myoblasts (passage numbers between 3 and 6) were cultured in Skeletal Muscle Growth Medium (Provitro) supplemented with 10% foetal bovine serum (Provitro) and kept in an incubator at 37 °C with 5% CO_2_. Differentiation was induced by treating cells with differentiation medium consisting of Dulbecco’s Modified Eagle’s Medium (DMEM) supplemented with GlutaMAX and 2% horse serum (all from Gibco^®^) for 7 days. The medium was changed every 2 days.

### RNA and DNA extraction and real time-PCR analysis

RNA isolation was performed by using High Pure RNA Isolation Kit (Roche Diagnostics) according to manufacturer’s recommendations. First-strand cDNA was synthesized from total RNA (0.7 μg) with oligonucleotide dT15 primers and random primers p(dN)6 by use of First Strand cDNA synthesis kit (Roche). Real time-PCRs were performed using Light Cycler 480 SYBR Green Master I (Roche) and were analysed by Light Cycler 480 SW 1.5 software (Roche). Oligonucleotide sequences used for PCR are listed in Table S1 indicating NCBI code. Primers were from Sigma (KiCqStart^®^ SYBR^®^ Green Primers) or designed using Primer3 software (http://primer3plus.com/cgi-bin/dev/primer3plus.cgi, last accessed March 16, 2017)^65^. Primer parameters were defined as follows: product size: 50–150 bp; primer size: 18–22 bp (opt: 20); primer Tm: 57–63 °C (opt: 60); primer GC%: 40–60%; maximum self-complementarity: 3–4. The Operon tool (http://www.operon.com/tools/oligo-analysis-tool.aspx, last accessed March 16, 2017) was utilized to check the putative primer-dimer formation. Amplification conditions consisted of 5 s of denaturation at 94 °C, 9 s of annealing at 55–60 °C, and 9 s of extension at 72 °C for each step for 45 cycles. The relative amount of all mRNA was calculated using comparative CT method*. Acidic Ribosomal Phosphoprotein P0* and *GAPDH* mRNA were used as the invariant controls for all studies^66^. Blast 2seq and Primerblast (NCBI: http://blast.ncbi.nlm.nih.gov/Blast.cgi, last accessed March 16, 2017) were used to check if primers could be aligned with sequences of other organisms or could detect other genes. For measurement of mtDNA copy number, total DNA isolation was performed by using QIAamp^®^ DNA Mini Kit (Qiagen). Real time-PCR conditions and primers for mtDNA analysis were performed according Venegas *et al*.^67^ and Lauritzen *et al*.^47^ with some modifications. *β2-microglobulin* gene was used as nuclear encoded gene for normalization of mtDNA content.

### Real-time metabolic assays

Metabolic pathways were assessed using the Seahorse XF^e^96 Extracellular Flux Analyzer (Seahorse Bioscience). Cells were plated in XF96‐well cell culture microplate (V3-PS, Seahorse Bioscience), at a density of 5 × 10^4^ cells per well for myoblasts and 8–12 × 10^3^ cells per well for myotubes. Differentiation was induced by same procedure as described above. Seahorse plates were pre-coated with 10 μg/cm^2^ of collagen I (Sigma) diluted in ultra-pure water (Sigma). To access if mitochondrial function could be restored by presence of laminin, cells plates were coated with 2 μg/cm^2^ of human rLaminin-211 (Biolamina) diluted in Ca^2+^/Mg^2+^ DPBS (Gibco), following the manufacturer’s instructions.

### Cellular bioenergetics

To measure mitochondrial function cells were placed in unbuffered Basal Assay Medium (Seahorse) supplemented with 10 mM glucose, 1 mM glutamine and 2 mM sodium pyruvate, pH 7.4 at 37 °C without CO_2_ for 1 h before the assay. Oxygen consumption rate (OCR) was monitored along time in repeated cycles of 3 minutes mix, 3 minutes measurement between injections of mitochondrial inhibitors (from Sigma): ATP synthase inhibitor oligomycin (final concentration: 5 μM), proton ionophore fluorocarbonyl cyanide phenylhydrazone (FCCP: 1 μM) and complex I inhibitor rotenone (1 μM). Bioenergetic and mitochondrial function parameters such as basal respiration, maximal respiration, ATP production, spare respiration, non-mitochondrial respiration and coupling efficiency were analysed. All cell conditions were analysed as ten biological replicates per patient and data were pooled to give average values for each condition. After the assays, plates were saved and protein concentrations for each well were measured as described above. Data collection and analyses were performed using Wave software - Version 2.2 (Seahorse Bioscience).

### Glycolytic function

At the day of experiment cells were placed in unbuffered Basal Assay Medium (Seahorse) supplemented with 1 mM glutamine pH 7.4 at 37 °C without CO_2_ for 1 h before the assay. Extracellular acidification rate (ECAR) was measured over time following injections of glucose (final concentration: 10 μM) to activate glycolysis, oligomycin (2.5 μM) to inhibit ATP synthase and 2-deoxy-D-glucose (50 mM) to inhibit glycolysis. Glycolytic function was analyzed based on parameters such as glycolytic capacity and reserve, glycolysis and non-glycolytic acidification.

### Fatty acid oxidation

To measure fatty acid oxidation (FAO) cell medium was changed one day before the assay to Substrate Limited Medium (DMEM (A14430–01-Gibco) supplemented with 0.5 mM glucose (Sigma), 1 mM glutamax (Gibco), 0.5 mM L-Carnitine (Sigma) and 1% FBS (Sigma)). At the day of experiment cells were placed in FAO assay medium (111 mM NaCl, 4.7 mM KCl, 1.25 mM CaCl_2_, 2 mM MgSO_4_, 1.2 mM NaH_2_PO_4_) supplemented with 2.5 mM glucose, 0.5 mM carnitine, and 5 mM HEPES, pH 7.4 at 37 °C. FAO assay was performed using XF Palmitate-BSA FAO Substrate (Seahorse Bioscience) according to manufacturer’s recommendations. Mitochondrial function was analyzed as described above with 3–7 biological replicates per patient and data were pooled to give average values for each condition.

### Mitochondrial membrane potential (Δψm)

Mitochondrial membrane potential (Δψm) was determined using BD™ MitoScreen kit (Becton-Dickinson Biosciences). Briefly, cells were trypsinized, harvested, and incubated with JC-1 (the cationic fluorescent dye probe 5,5′,6,6′-tetrachloro-1,1′,3,3′-tetraethylbenzimidazolcarbocyanine iodide) according to manufacturer’s specifications. The analysis of JC-1 was performed on Becton Dickinson FACS LSR II SORP. JC1 was excited by 488 nm 100 mW Blue laser and detected on Alexa Fluor 488/FITC channel with 502LP dichroic mirror and 525/50 bandpass filter. Analysis was done on FACS diva software version 6.2. We present results as percentage of polarized mitochondria as functional mitochondria.

### Protein extraction and quantification

For enzymatic assays, protein lysates were obtained at 4 °C by incubating cell pellets from myoblasts and myotubes in lysis buffer (50 mM HEPES pH 7.5, 5 mM EDTA pH 8, 150 mM NaCl and 0.1% CHAPS) for 30 minutes with vortexing in 10 minutes intervals. Lysates were centrifuged at 13.000 rpm for 17 minutes at 4 °C. The protein concentrations were determined by method of the bicinchoninic acid with the commercial BCA™ Protein Assay kit (Thermo Scientific).

### Cell cycle

The cell cycle phase distribution was determined using BD™ CycleTEST^TM^ Plus DNA Reagent kit (Becton-Dickinson Biosciences) according to manufacturer’s instructions. The analysis of PI cell cycle was performed on Becton Dickinson FACS Aria III. PI was excited by 561 nm 50 mW Yellow-Green Laser and detected on PI/PE-TxRed channel with 595LP dichroic mirror and 610/20 bandpass filter. Analysis was done on FACS diva software version 7.0.

### Caspase-3 enzymatic assay

Caspase-3 activity was measured using the Caspase-3 Colorimetric Activity Assay Kit (Millipore). Protein samples were incubated for 1 h at 37 °C with Ac-DEVD-p-nitroaniline (DEVD-pNA) in assay buffer (provided in the kit) according to manufacturer’s instructions. Active caspase-3 from the protein samples cleaves pNA from DEVD-pNA. The free pNA was quantified measuring the absorbance at 400 nm using a microplate reader (Wallac Victor 1420, Perkin Elmer).

### 20S Proteasome enzymatic assay

20S proteasome activity was measured using the 20S Proteasome Activity Assay Kit (Millipore), which detects the chymotrypsin-like activity by monitoring amido-4-methylcoumarin (AMC) release from the synthetic peptide substrate LLVY-AMC. Protein samples were added to a black 96 well plate and incubated with Suc-LLVY-7-amino-4-methylcoumarin (LLVY-AMC) in assay buffer for 1 h at 37 °C according to manufacturer’s instructions. The free AMC peptide was measured by a fluorimeter (Wallac Victor 1420, Perkin Elmer) using an excitation wavelength of 380 nm excitation and an emission wavelength of 460 nm.

### Statistical analysis

All data are shown as mean ± S.E.M. Levene test was used to assess the homogeneity of variances among groups. Statistical analysis of the data was performed by means of one-way analysis of variance (ANOVA) with Duncan post hoc test. For laminin-211 rescue experiment, t test was used to compare differences between myotubes from MDC1A patients with and without laminin-211 coating. *p* < 0.05 values were considered as statistically significantly different. Letters **a**, **b** and **c** were used to express the differences among groups and columns with the same letter are not statistically significantly different from each other. For each parameter, **b** and **c** represent a significant difference from control **a**; and **c** is used when Surf1-LS is significantly different from MDC1A and control groups.

## Additional Information

**How to cite this article:** Fontes-Oliveira, C. C. *et al*. Bioenergetic Impairment in Congenital Muscular Dystrophy Type 1A and Leigh Syndrome Muscle Cells. *Sci. Rep.* **7**, 45272; doi: 10.1038/srep45272 (2017).

**Publisher's note:** Springer Nature remains neutral with regard to jurisdictional claims in published maps and institutional affiliations.

## Supplementary Material

Supplementary Information
