# Supplementary material for: Bioenergetic Impairment in Congenital Muscular Dystrophy Type 1A and Leigh Syndrome Muscle Cells
Source: Sci Rep. 2017 Apr 3;7:45272. doi: 10.1038/srep45272 (PMC5377256; doi:10.1038/srep45272)
Supplement: Supplementary Information [file srep45272-s1.pdf]

Supplementary Information

Bioenergetic Impairment in Congenital Muscular Dystrophy Type 1A and Leigh Syndrome Muscle Cells

Cibely C. Fontes-Oliveira, Maarten Steinz, Peter Schneiderat, Hindrik Mulder and Madeleine Durbeej

Table S1. Primers Sequences used for Real Time-PCR analysis

Symbol	Left (5´- 3´)	Right (5´- 3´)	NCBI Code	Source
PSMA2	ACCGAGAAAAAGCAGAAATCCA	ATGGACGAACACCACCTGAC	NM_002787.4	Designed
USP19	AGCGGCACAAGATGAGAAAT	ACGGGTCAAAAGTGATGGAG	NM_001199160.1	Designed
PGC1α	AAGGTCCCCAGGCAGTAGAT	TTCAGACTCCCGCTTCTCAT	NM_013261.3	Designed
MURF1	CCTGAGAGCCATTGACTTTGG	CTTCCCTTCTGTGGACTCTTCCT	NM_032588.3	Designed
ANT1	GCTGGTGTCCTATCCGTTTG	CAGTCAAGTGTCCCCGTGTA	NM_001151.3	Designed
MAFBx	TGTGCGATGTTACCCAAGAA	GGTGAAAGTGAGACGGAGCA	NM_058229.3	Designed
P0	ACATCTCCCCCTTCTCCTTCGG	GTTGCGGACACCCTCCAGAAAG	NM_001002.3	Designed
GAPDH	ACAGTCAGCCGCATCTTCTTT	CCCAATACGACCAAATCCGTTG	NM_002046.5	Designed
ATP5I	CAATTACCTAAAACCTCGGG	GTTTCAGTTCATCCTGCTTC	NM_007100.3	KiCqStart®
IDH3A	CACCCATCTATGAATTTACTGC	AATCACATGCTCAATTCCAC	NM_005530.2	KiCqStart®
NDUFA8	AAGGTCACCAAAGTGAAAAC	TTTTCATCAGTCGTTGTCTG	NM_014222.2	KiCqStart®
NDUFS2	GATGTTTGAGTTCTACGAGC	GATTTCGCCAGATCCTATTG	NM_004550.4	KiCqStart®
PDHA1	ATTCGAAGCTTACAACATGG	AGGATATCCATTCCATCCAC	NM_000284.3	KiCqStart®
PFKM	AGGACCAGACAGATTTTGAG	GATCAGGTAATCTATTCCCCTC	NM_001166686.1	KiCqStart®
PYGM	CATTGTCAATATGCTCATGC	CTCCTGGCATTTAATGTAGTC	NM_005609.2	KiCqStart®
CASP3	AAAGCACTGGAATGACATC	CGCATCAATTCCACAATTTC	NM_004346.3	KiCqStart®
SDHA	AGCATGCAGAAGTCAATG	ATTTTCCCACAACCTTCTTG	NM_004168.3	KiCqStart®
PGAM2	CTACTACAACTCCATTAGCAAG	TTGATCTGGGGAACAATCTC	NM_000290.3	KiCqStart®
mtDNA tRNALeu(UUR)	CACCCAAGAACAGGGTTTGT	TGGCCATGGGTATGTTGTTA	NC_012920.1	Venegas et al. [61]
nDNA β2M	TGCTGTCTCCATGTTTGATGTATCT	TCTCTGCTCCCCACCTCTAAGT	NM_004048.2	Venegas et al. [61]

Abbreviations: NCBI: National Center for Biotechnology Information (http://www.ncbi.nlm.nih.gov/nuccore/). *PSMA2*: proteasome subunit alpha 2; *USP19*: ubiquitin specific peptidase 19; *PGC-1α*: Peroxisome proliferator-activated receptor gamma coactivator 1-alpha; *MURF1*: muscle-specific RING finger protein 1; *ANT1*: adenine nucleotide translocator 1; *MAFBx*: muscle atrophy F-box protein; *P0*: Acidic Ribosomal Phosphoprotein P0; *GAPDH*: glyceraldehyde-3-phosphate dehydrogenase; *ATP5I*: ATP synthase, H+ transporting, mitochondrial F0 complex subunit E; *IDH3A*: isocitrate dehydrogenase 3*; NDUFA8*: NADH:ubiquinone oxidoreductase subunit A8; *NDUFS2*: NADH dehydrogenase (ubiquinone) Fe-S protein 2; *PDHA1:* pyruvate dehydrogenase (lipoamide) alpha 1; *PFKM:* phosphofructokinase; *PYGM:* glycogen phosphorylase, muscle form; *CASP3*: caspase-3; *SDHA*: succinate dehydrogenase complex flavoprotein subunit A; *PGAM2:* phosphoglycerate mutase 2; *mtDNA tRNALeu(UUR)*: mitochondrially encoded tRNA leucine 1 (UUA/G) *β2M*: beta-2-microglobulin; Source: Designed using primer3 software, purchased by Sigma (KiCqStart predesigned primers) and Venegas et al[61].

Figure S1. Pattern of *mt-COX1* expression in MDC1A and Surf1-LS myogenic cells.

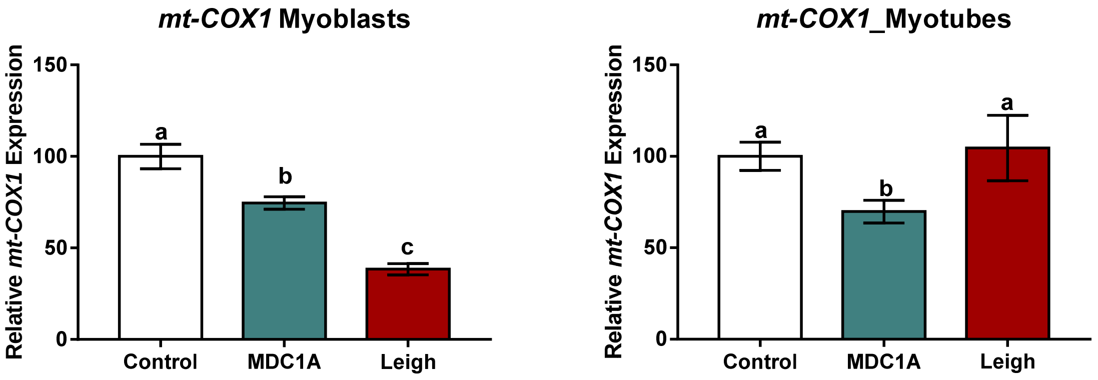

Results represent differences of gene expression in human muscle cells (myoblasts and myotubes as indicated) among control subjects, MDC1A and Surf1-LS patients groups. Statistical significance was assessed by one-way ANOVA followed by Duncan's post hoc test. Different letters (a, b and c) indicate significant differences ($p < 0.05$) among the groups.
